# Case report: Management of recurrent pupillary optic capture with sutureless surgical technique using 7–0 polypropylene flange

**DOI:** 10.3389/fmed.2024.1367905

**Published:** 2024-02-22

**Authors:** Dong Hyeon Kim, Da Ru Chi Moon, Yong Koo Kang, Dong Ho Park, Jae Pil Shin, Dai Woo Kim, Jae Rock Do

**Affiliations:** ^1^Department of Ophthalmology, School of Medicine, Kyungpook National University, Kyungpook National University Hospital, Daegu, Republic of Korea; ^2^Nune Eye Hospital, Daegu, Republic of Korea

**Keywords:** flanged intraocular lens fixation, intraocular lens dislocation, pupillary optic capture, 7–0 polypropylene flange, sutureless

## Abstract

**Background:**

To report a novel surgical technique for recurrent pupillary optic capture after flanged intraocular lens (IOL) fixation.

**Methods:**

In this retrospective case series, we detail our use of two parallel 7–0 polypropylene sutures passed between the iris plane and the optic of scleral-fixated IOL to address pupillary optic capture. Flanges were created using ophthalmic cautery to secure it to the sclera without suture.

**Results:**

Two eyes with pupillary optic capture underwent a sutureless surgical technique using 7–0 polypropylene flanges. No recurrences of pupillary optic capture were observed during the 1-year follow-up.

**Conclusion:**

Our sutureless surgical technique using a 7–0 polypropylene flange was an effective, efficient, and less invasive approach for treating recurrent pupillary optic capture.

## Background

Secondary flanged intraocular lens (IOL) implantation can be performed in eyes with a capsular defect. Various surgical techniques have been utilized for secondary IOL implantation, including sutured scleral fixation (1). Recently, Yamane et al. (2) introduced a novel technique called flanged IOL fixation, which has demonstrated shorter operative times than sutured scleral fixation techniques with comparable clinical outcomes (3).

Pupillary optic capture of IOL is a common complication of sutured scleral fixation of IOL implantation, with an incidence rate ranging from 7.9% to 23.0% (4, 5). Similarly, in cases with flanged IOL fixation, incidence rates of pupillary optic capture have ranged from 8% to 38.9% (2, 6, 7). Pupillary optic capture of IOL leads to blurred vision and photophobia and can cause chronic uveitis, macular edema, and secondary glaucoma.

Most cases of optic capture have been managed in the office by pushing the optic posterior to the iris using a 30-guage needle (8). However, repeated recurrence of pupillary optic capture despite these techniques may warrant surgical interventions, including IOL repositioning (9–11) and exchange (10).

Recently, Lin et al. (12) reported on a technique for IOL repositioning following pupillary optic capture using a 10–0 nylon suture between the iris plane and IOL optic. However, this technique requires conjunctival dissection, with several studies reporting the degradation or breakage of the 10–0 thread suture (13–15). Therefore, we aimed to introduce a new technique for sutureless IOL repositioning following pupillary optic capture using the 7–0 polypropylene flange.

## Methods

### 7–0 polypropylene flange technique

**Figure 1:**
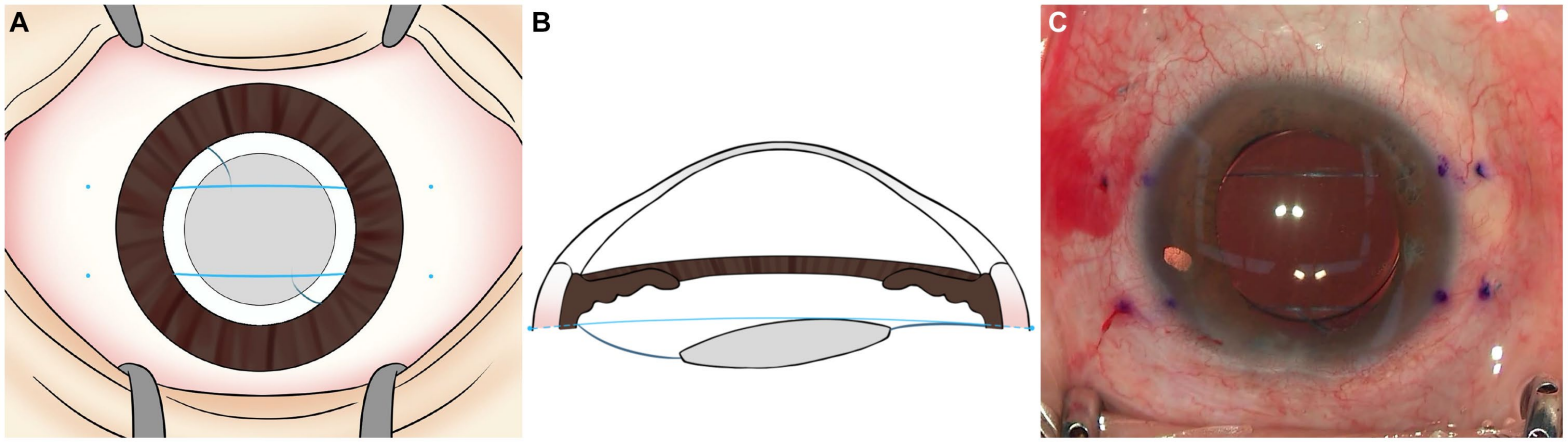
**(A,B)** Schematic image and **(C)** intraoperative image of 7–0 polypropylene flange technique. 7–0 polypropylene sutures are inserted 2.0 mm posterior to the limbus. A pair of sutures is placed between the posterior surface of iris and the optic of the IOL, parallel to each other and perpendicular to the iris edge capturing the IOL.

Three days before the surgery, topical 1.5% levofloxacin eye drops were applied four times a day. Preoperative preparation was conducted in the operating room using 5% povidone-iodine. We designed a surgical technique wherein a pair of parallel 7–0 polypropylene sutures is positioned between the posterior surface of iris and the optic of the IOL to separate the iris plane from the IOL. The sutures would run perpendicular to the iris edge, capturing the IOL with a 4-mm distance between the two sutures (Figure 1).

**Figure 2:**
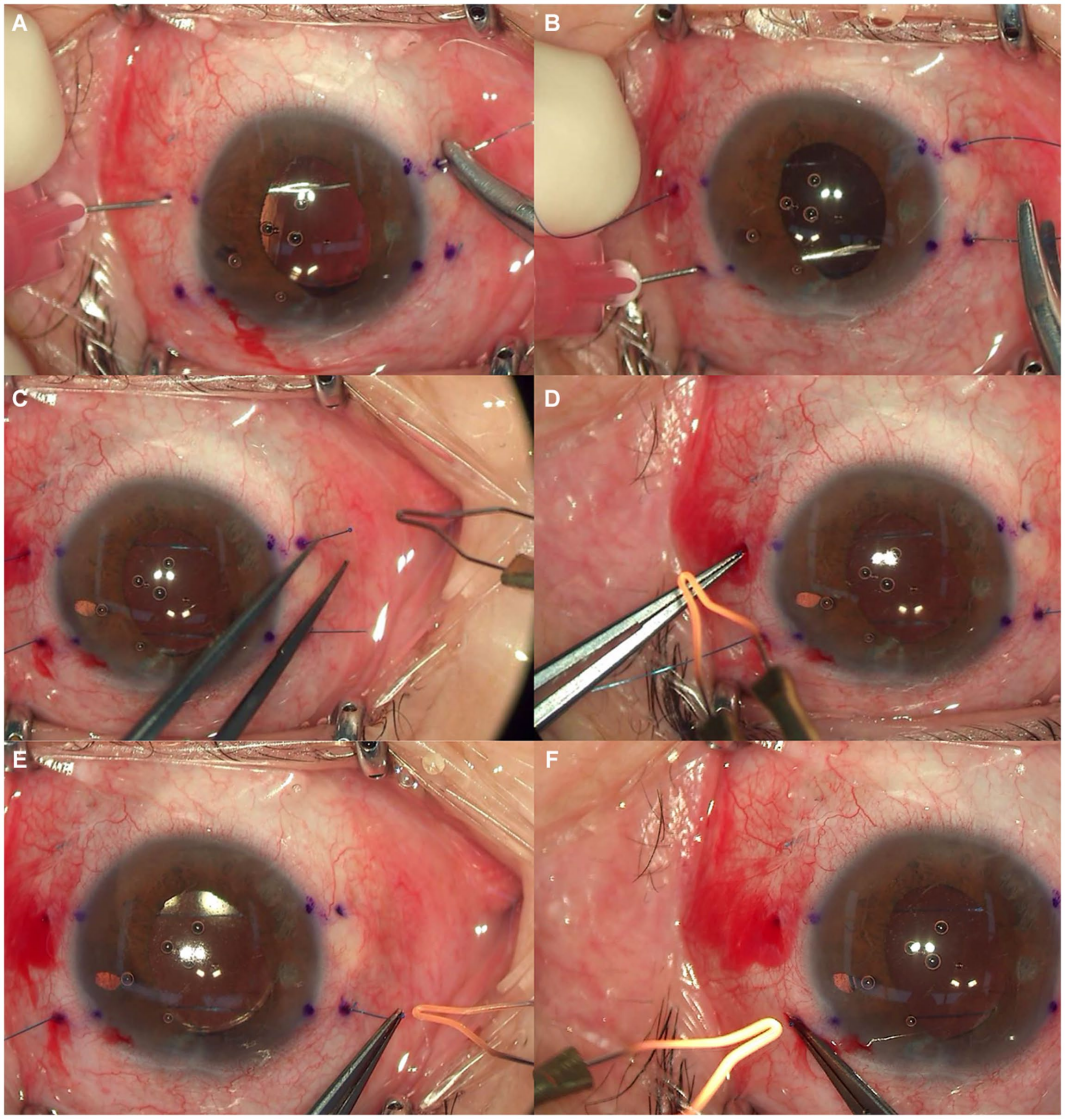
**(A)** 7–0 polypropylene suture inserted through the temporal sclera, passed through the posterior surface of the iris plane, and externalized through nasal sclera. **(B)** The other suture inserted and externalized in the same manner. **(C–F)** Two temporal flanges were created via ophthalmic cautery and inserted into the scleral tunnel. Thereafter, two nasal flanges were created and then placed inro the scleral tunnels.

After subtenon anesthesia, a 7–0 polypropylene suture with a straight needle is inserted through the temporal sclera, 2.0 mm from the limbus, and passed through the posterior surface of the iris plane without making a conjunctival incision. Using a 29-gauge needle, a nasal sclerotomy is made 2.0 mm from the limbus to the space between the iris and the IOL. The captured IOL is released by gently pushing the optic behind the iris using either the 29-gauge needle or the 7–0 polypropylene suture. After docking the 7–0 polypropylene suture into the 29-gauge needle in front of the IOL optic, the suture is then externalized, and both ends are cut. The other 7–0 polypropylene suture is inserted again and extracted from the anterior chamber in the same manner, after which both ends are cut. The temporal flange is created via ophthalmic cautery (Accu-Temp Cautery; Beaver Visitec, Waltham, MA, United States) and inserted inside the scleral tunnel. After slightly pulling the nasal end of suture to maintain suture tension, the nasal suture is cut approximately 2 mm from its base. The nasal flange is created by ophthalmic cautery and then placed in the scleral tunnel. The two flanges of the other suture are created in the same manner (Figure 2) (Supplementary Video S1, Supplemental Digital Content).

## Results

### Case 1

**Figure 3:**
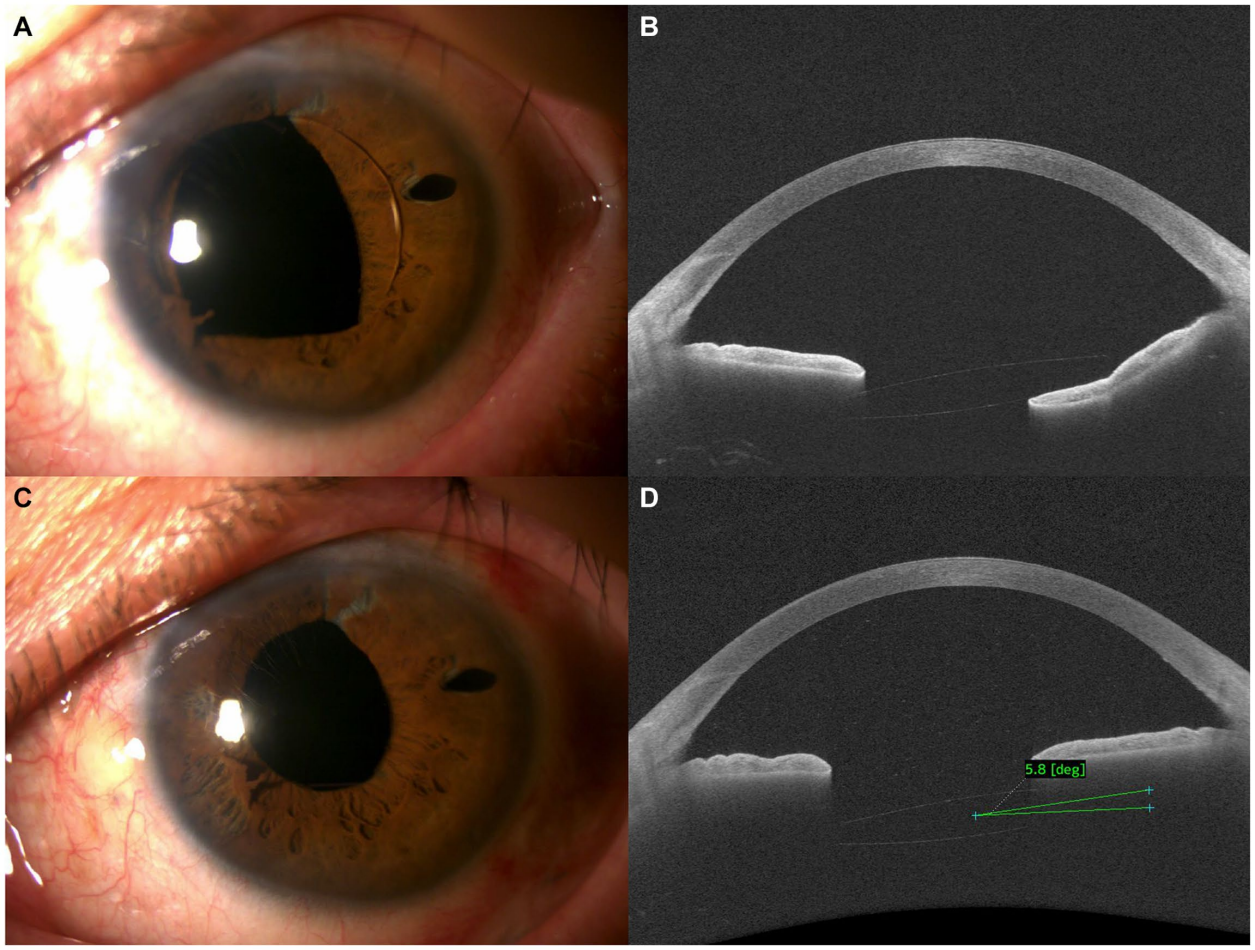
**(A,B)** Preoperative anterior segment **(A)** and anterior segment optical coherence tomography (AS-OCT) images **(B)** demonstrating pupillary optic capture of the intraocular lens (IOL) after flanged IOL fixation. **(C,D)** Postoperative anterior segment **(C)** and AS-OCT images **(D)** demonstrating resolution of pupillary optic capture after IOL repositioning with a 7–0 polypropylene flange.

A 62-year-old male was referred for rhegmatogenous retinal detachment with a superior giant tear and lens subluxation in the left eye following blunt trauma. In response, we performed phacoemulsification, pars plana vitrectomy, flanged IOL fixation, and silicone oil injection. Given that the retina remained well attached 3 months after surgery, we subsequently performed silicone oil removal. One week after silicone oil removal, the patient had an uncorrected visual acuity of 20/20 (−0.25 Diopter (D) sphere (sph) −0.5 D cylinder (cyl) × 75°) and retina remained well attached with a well-positioned scleral-fixated IOL. However, 3 weeks after silicone oil removal, the patient complained of blurred vision, photopsia, and dull pain and was diagnosed with pupillary optic capture of the scleral-fixated IOL (Figure 3A). Given the patent iridectomy site, we repositioned the optic back into the posterior chamber using a 30-guage needle and utilized pilocarpine 2% eye drops to prevent recurrence of pupillary optic capture. However, recurrence was noted 1 week after repositioning, accompanied by an increase in intraocular pressure (IOP) to 38 mmHg and a decrease in visual acuity to 20/50 (+0.25 D sph −1.75 D cyl × 115°). anterior segment optical coherence tomography (AS-OCT) showed a 9.7° tilt in the IOL (Figure 3B). In response, IOL repositioning using a 7–0 polypropylene flange was performed on the same day. Considering that the axis of pupillary optic capture was horizontal, we also performed suturing in the horizontal axis direction. The operative time was about 13 min, and there were no postoperative complications. One week after IOL repositioning with a 7–0 polypropylene flange, pupillary optic capture was successfully relieved (Figure 3C). The visual acuity improved to 20/20, refraction had returned (−0.25 D sph −0.5 D cyl × 75°), IOP was normalized to 12 mmHg, and the IOL tilt decreased to 5.8° on AS-OCT (Figure 3D). No recurrence was observed during a 1-year follow-up period.

### Case 2

A 59-year-old male was referred to our clinic for IOL dislocation after blunt trauma. The patient underwent vitrectomy, IOL removal, and flanged IOL fixation. Three weeks after surgery, the patient reported a decrease in his visual acuity to 20/63 (−2.75 D sph −0.55 D cyl × 160°), and dull pain in his right eye. Upon slit lamp examination, pupillary optic capture was diagnosed. we then repositioned the optic back into the posterior chamber using a 30-guage needle. However, recurrence was noted in the horizontal axis, for which IOL repositioning using a 7–0 polypropylene flange in the horizontal axis direction was subsequently performed. The operative time was about 10 min. Vitreous hemorrhage was observed the day after surgery. However, it resolved by the 7 days after the surgery. Following this procedure, the patient achieved a best-corrected visual acuity of 20/20 (−0.5 D sph −0.25 D cyl × 145°). No recurrence was observed during a 1-year follow-up period.

## Discussion

Pupillary optic capture after scleral IOL fixation is a relatively common postoperative complication (2, 4–7). Although the mechanism for pupillary optic capture has yet to be fully elucidated, several possible causes for such a complication after scleral IOL fixation have been reported, such as anterior chamber depth, axial length, and IOL tilt (16–19).

Pupilloplasty has been suggested as an effective surgical procedure for the treatment of pupillary optic capture by narrowing the pupil size (18, 20). However, in cases of pupillary optic capture with severe IOL tilt, iris–IOL contact can persist after pupilloplasty, causing pigment dispersion that may accumulate in the trabecular meshwork, precipitating pigmentary glaucoma (21–23). It also decreases iris regulatory function, making it difficult to observe the peripheral retina in patients with retinal disorders (Supplementary Table S1).

Lin et al. (12) reported a surgical technique using a 10–0 nylon suture between the iris plane and IOL optic for the treatment of pupillary optic capture. This technique is more suitable for patients with retinal disorders in that iris regulatory function is preserved after surgery. However, degradation or breakage of the 10–0 thread suture has been reported (14).

Our surgical technique using a 7–0 polypropylene flange does not require conjunctival resection and eliminates the need for suturing. Moreover, a flange could mitigate the risk of suture degradation or breakage, which can occur with the suturing technique. Additionally, this technique can preserve the iris regulatory function after surgery, making it possible to do the dilated retinal examination.

Kokame et al. (8) suggested that in-office management was a good option for managing optic capture. Although this procedure can be performed in the office without discomfort or significant complications, pupillary optic capture may recur after the procedure. In our cases, pupillary optic capture recurred despite implementing in-office management, possibly attributed to the previous blunt trauma history. A previous study reported that trauma can lead to zonular dialysis, iris retraction, iridodonesis, and angle recession, which increase the risk of pupillary optic capture (24).

In our cases, IOL tilt along the axis of pupillary optic capture decreased after surgery. Makoto et al. (25) reported no changes after bridging sutures using a 10–0 polypropylene suture with a scleral tunnel of 1.5 mm posterior to the limbus. This difference may be attributed to our surgical procedure, which uses thicker 7–0 polypropylene suture flanges with a scleral tunnel of 2 mm posterior to the limbus. These could have induced a stronger pushing force onto the anterior part of the tilted IOL posterior and lowering the potential risk of pigment dispersion due to iris–suture contact by maintaining a sufficient distance between them.

The IOLs that underwent pupillary optic capture were ZA9003 and AR40e, which had an optic diameter of 6 mm. Therefore, we established a 4-mm distance between the two sutures. The distance between the two sutures might be important given that an excessively short distance can affect the patient’s visual field, whereas an excessively long distance may prevent the sutures from being able to appropriately support the IOL posteriorly. Patients who underwent this management did not have any symptoms of visual impairment. Additionally, no visual field impairments were observed during the visual field test.

Despite of relatively easy learning curve and short operative time, potential complications may occur after this technique. Vitreous hemorrhage was observed in 1 case during the surgery when the needle encountered the ciliary body while passing the 7–0 polypropylene suture. Fortunately, the bleeding was promptly controlled by increasing the IOP with a BSS injection. To mitigate this complication, the authors recommend the following tips: (1) Maintain a distance of 2 mm or more between the sclerotomy site and limbus; (2) Ensure sufficient IOP with a BSS injection before creating the sclerotomy; (3) when creating the sclerotomy, pass the 7–0 polypropylene suture and needle perpendicular to the sclera to avoid touch the ciliary body.

Our study has several limitations, including its small sample size. Additionally, despite the numerous reports on haptic flange stability in flanged IOL fixation (26), additional research is needed to investigate the long-term stability of the suture flange. Third, given our inability to precisely quantify the astigmatism change after surgery, we were unable to assess corneal curvature and postoperative astigmatic changes due to the decreased IOL tilt. Further research is needed to investigate whether decreasing the IOL tilt may be associated with changes in refractive astigmatism. Furthermore, we conducted the surgical technique only for cases with pupillary optic capture occurring after flanged IOL fixation. With the mechanism of this surgical technique, it is anticipated that it can also be applied to cases of pupillary optic capture after sutured scleral fixation, further research will be needed.

## Conclusion

Our sutureless surgical technique using a 7–0 polypropylene suture flange can be an efficient alternative for treating recalcitrant pupillary optic capture.

## Data availability statement

The original contributions presented in the study are included in the article/Supplementary material, further inquiries can be directed to the corresponding author.

## Ethics statement

The studies involving humans were approved by Institutional Review Board of Kyungpook National University Hospital (IRB No. 2023-12-026). The studies were conducted in accordance with the local legislation and institutional requirements. Written informed consent for participation in this study was provided by the participants’ legal guardians/next of kin. Written informed consent was obtained from the individual(s) for the publication of any potentially identifiable images or data included in this article.

## Author contributions

DHK: Writing – original draft, Writing – review & editing. DM: Conceptualization, Data curation, Writing – review & editing. YK: Conceptualization, Data curation, Methodology, Writing – review & editing. DP: Writing – original draft, Writing – review & editing. JS: Writing – review & editing. DWK: Writing – review & editing. JD: Conceptualization, Data curation, Formal analysis, Funding acquisition, Investigation, Methodology, Project administration, Resources, Software, Supervision, Validation, Visualization, Writing – original draft, Writing – review & editing.
