# Supplementary material for: Case report: Management of recurrent pupillary optic capture with sutureless surgical technique using 7–0 polypropylene flange
Source: Front Med (Lausanne). 2024 Feb 22;11:1367905. doi: 10.3389/fmed.2024.1367905 (PMC10918005; doi:10.3389/fmed.2024.1367905)
Supplement: Supplementary file 1 [file Table_1.DOCX]

Supplementary Material

**Supplementary Table 1.** Advantages and disadvantages of various surgical techniques for managing pupillary optic capture after scleral fixation

| **Technique type** | **Method** | **Advantages** | **Disadvantages** |
| --- | --- | --- | --- |
| In-office management | Pushing the captured optic posterior to the iris using a 30-gauge needle | Easily performed using a slit lamp  Short operative time | High recurrence rate |
| Pupilloplasty | Narrowing the pupil size with an iris suture after releasing the optic capture | Low recurrence rate | Long operative time  Difficulty observing the peripheral retina after surgery |
| IOL repositioning using 10–0 nylon sutures | Placing 10–0 nylon sutures between the iris plane and IOL optic after releasing the optic capture | Preserved iris regulatory function | Risk of suture degradation or breakage |
| IOL repositioning using 7–0 polypropylene flanges | Placing 7–0 polypropylene flanges between the iris plane and the IOL optic after releasing the optic capture | Preserved iris regulatory function  Short operative time  No need for conjunctival incision  Sutureless technique | Learning curve for making flanges |

IOL = intraocular lens.
